# Unequal relief from the double burden: job quality, education, and women’s time poverty in India—evidence from NSS time use survey 2024

**DOI:** 10.3389/fsoc.2026.1862733

**Published:** 2026-06-22

**Authors:** Mayadhar Sethy, Sandhya R. Mahapatro, Udaya Shankar Mishra

**Affiliations:** 1 Nabakrushna Choudhury Centre for Development Studies, Bhubaneswar, Odisha, India; 2 International Institute for Population Sciences, Mumbai, Maharashtra, India

**Keywords:** double burden, instrumental variables, job quality index, time poverty, time use survey

## Abstract

**Background:**

Women’s increasing participation in paid employment in India has not eliminated the persistent “double burden” of paid and unpaid work. Existing studies largely rely on binary employment measures and overlook heterogeneity in job quality and education. This study examines how multidimensional job quality and educational attainment jointly shape women’s unpaid work and time poverty in India.

**Methods:**

Using nationally representative data from the NSS Time Use Survey 2024 (101,234 women aged 15–59 years), we construct a multidimensional Job Quality Index (JQI) using Principal Component Analysis incorporating employment formality, income adequacy, social security coverage, and decent working hours. We develop a Circular Multidimensional Time Poverty Index (CMTPI) as an exploratory visualization framework. Empirical strategy combines descriptive intersectional analysis, multivariate OLS regression with interaction effects, and instrumental variable (IV-2SLS) estimation.

**Results:**

Employed women perform 9.6 h of total daily work and undertake nearly five times more unpaid work than men. A “squeezed middle” emerges: secondary-educated women in low-quality jobs experience the highest total workload (≈559 min). A one-standard-deviation increase in job quality is associated with 42–58 min reduction in unpaid work. Education amplifies this association, with graduate women gaining approximately 67 min of relief compared with minimal reduction among illiterate women. Intersectional disparities persist across urban–rural, caste, and income groups.

**Conclusion:**

Employment alone does not reduce women’s unpaid work; only decent jobs combined with education alleviate the double burden. Policies should prioritize improving job quality, expanding childcare and time-saving infrastructure, and promoting redistribution of unpaid work.

## 1 Introduction

The integration of women into formal labor markets is widely framed as a driver of economic growth, poverty reduction, and gender equality (Kabeer, 2012; Duflo, 2012; Kabeer, 2021). International organizations and development agencies increasingly promote female labor force participation (FLFP) as a critical indicator of progress toward Sustainable Development Goal 5 (gender equality) and Goal 8 (decent work). However, this optimistic narrative increasingly conflicts with persistent realities across low- and middle-income countries. Women’s entry into paid work rarely produces a proportional reduction in unpaid domestic labor (Charmes, 2019; Folbre, 2018). Instead, women accumulate paid employment atop existing household responsibilities, generating chronic “time poverty” that undermines well-being, health, productivity, and economic advancement (Hochschild and Machung, 2012; Hirway, 2015). This phenomenon, commonly conceptualized as the “double burden,” remains one of the most persistent structural barriers to gender equality in the Global South (Seguino, 2000).

In India, the double burden is particularly acute. Despite sustained economic growth over the past three decades, female labor force participation remains persistently low at approximately 25–30%, among the lowest within the G20 economies (World Bank, 2023). Existing scholarship documents stagnation and even decline in urban female employment participation despite rising educational attainment (Klasen and Pieters, 2015; Mehrotra and Parida, 2017). Evidence from the NSS Time Use Survey 2024 reveals stark gender asymmetries in daily labor allocation: employed women spend over 9.5 h per day on combined paid and unpaid work, substantially exceeding men’s average workload of approximately 7.2 h (NSO, 2024). Women also perform nearly five times more unpaid domestic and care labor than men. This severe time squeeze channels women into informal, flexible, and low-paying employment arrangements, perpetuates gender wage disparities, restricts career mobility, and negatively affects physical and mental health outcomes (Mahapatro and Sethy, 2025; Srija and Vijay, 2020; Samantroy, 2022).

Despite growing attention to unpaid work and time poverty, three major analytical gaps continue to limit existing research.

- First, the literature overwhelmingly treats employment as binary, obscuring profound heterogeneity in job quality—security, income adequacy, working conditions, and social protections (International Labour Organization, 2018; Kalleberg, 2011).
- Second, although education is recognized as a key moderator of labor market outcomes (Kingdon and Unni, 2001), its interaction with multidimensional job quality remains underexplored in time-use research.
- Third, methodological challenges—particularly potential endogeneity between employment choice and domestic responsibilities—are routinely ignored (Mullainathan and Shafir, 2013; Field et al., 2021).

This study addresses these gaps through three key contributions. First, we construct a multidimensional Job Quality Index (JQI) that captures employment formality, income adequacy, social security coverage, and decent working hours, thereby moving beyond simplistic binary employment measures. Second, we develop a Circular Multidimensional Time Poverty Index (CMTPI) as an exploratory visualization framework that enables intersectional comparisons across education, caste, income, and regional dimensions. Third, we employ an instrumental variable estimation strategy using district-level female labor force participation as an exogenous source of variation to address potential endogeneity between job quality and unpaid work.

The study examines four central hypotheses:

*H1*: Employed women experience lower unpaid work than non-employed women, conditional on job quality.

*H2*: The negative relationship between employment and unpaid work is stronger among women engaged in high-quality jobs.

*H3*: Higher educational attainment amplifies the negative effect of job quality on unpaid work and time poverty.

*H4*: The burden-reducing effects of high-quality employment are unevenly distributed across caste, class, and geographic groups.

Using nationally representative data from the NSS Time Use Survey 2024 comprising 101,234 women aged 15–59 years, the study demonstrates that employment alone does not automatically reduce women’s time poverty. Instead, the benefits of employment depend critically on the quality of work and women’s educational position. The findings identify a distinct “squeezed middle” group—secondary-educated women engaged in low-quality employment—who experience the highest total workload, exceeding both less-educated women and graduate women. Improvements in job quality are associated with substantial reductions in unpaid work, although these gains remain highly unequal across caste and income groups.

The findings challenge conventional policy narratives that equate employment with empowerment. Expanding female labor force participation without simultaneous improvements in job quality, care infrastructure, and redistribution of unpaid labor may intensify rather than alleviate women’s time poverty. The study therefore argues for integrated policy interventions combining decent work creation, childcare expansion, public infrastructure investment, and gender norm transformation aimed at redistributing unpaid domestic work.

## 2 Literature review and conceptual framework

### 2.1 Theoretical foundations

This study is anchored in three interlocking theoretical frameworks:

Feminist economics and double burden theory document the historical inertia in the gendered division of labor, where women’s entry into paid work is added to, rather than substituted for, pre-existing unpaid domestic responsibilities (Hochschild and Machung, 2012; Folbre, 1994). This creates a systemic “time squeeze” with documented effects on health and productivity (Hirway, 2015; Doss, 2013).

Household bargaining models provide micro-foundations for understanding variations in the double burden. Rejecting the unitary household assumption, these models posit that intra-household allocations depend on members’ relative bargaining power derived from earnings, assets, and socially sanctioned alternatives (Manser and Brown, 1980; Lundberg and Pollak, 1993). However, Agarwal (1997) concept of “bargaining with patriarchy” notes that social norms constrain bargaining even when women possess economic resources (Bittman et al., 2003).

The capabilities approach (Sen, 1999; Nussbaum, 2000) extends the analysis from time allocation to human welfare, agency, and freedom. The double burden constrains women’s real freedoms to live valued lives, making time poverty a fundamental deprivation (Bianchi et al., 2000; Robeyns, 2003).

### 2.2 Labor market segmentation and precarious employment

The literature on labor market segmentation and precarious employment is highly relevant to this study. Dual labor market theory distinguishes primary (stable, well-paid, with benefits) and secondary (unstable, low-paid, without protections) sectors (Doeringer and Piore, 1971). Indian women are disproportionately concentrated in the secondary sector, where informal and precarious employment prevails (Berg and Kalleberg, 2021). This segmentation reinforces the double burden because precarious jobs lack the income stability and bargaining power needed to reallocate domestic work (Bescond et al., 2003; Green, 2006).

### 2.3 Empirical evidence and research gaps

Cross-national time-use studies indicate that while men’s domestic contributions have risen modestly in high-income countries, women continue to perform 2–5 times more unpaid labor across all contexts (International Labour Organization, 2018; Hook, 2010). In India, Klasen and Pieters (2015) highlight the stagnation of urban FLFP despite economic growth. Chattopadhyay (2021) and Gupta and Ghosh (2021) confirm that employed women work significantly longer total hours than men.

Education emerges as a key mediator. Kingdon and Unni (2001) show that education increases women’s probability of formal employment, while Afridi et al. (2018) find that educated women delay marriage and childbearing. Recent studies have examined unpaid work through time-use surveys: Srija and Vijay (2020) analyze FLFP patterns; Samantroy (2022) examines valuation of unpaid work; Sengupta et al. (2017) explore the “double boon” concept; and Gupta and Pattanaik (2025) investigate the relationship between time and work for Indian women (Crenshaw, 1991).

Despite extensive documentation, three critical gaps remain: (Agarwal, 1997) employment is rarely treated as a multidimensional construct; (Barker et al., 2020) intersectionality remains limited in time-use research; (Berg and Kalleberg, 2021) endogeneity is routinely ignored (Bridges et al., 2022; Rao, 2014).

## 3 Data and methodology

### 3.1 Data source and sample

This study uses unit-level data from India’s first dedicated Time Use Survey (TUS) conducted by the National Statistical Office (NSO) between January and December 2024 (NSO, 2024). The survey employs a stratified two-stage sampling design, ensuring national representativeness. Data were collected using Computer-Assisted Personal Interviewing (CAPI) with 24-h activity diaries in 30-min intervals, classified according to ICATUS 2016 (United Nations, 2016).

The analytical sample is restricted to women aged 15–59 years. Excluding women aged 60 + years, girls under 15 years, and observations with missing data (<2% of the sample), the final weighted sample comprises 101,234 women.

Supplementary Table 1 provides the ICATUS classification codes for unpaid work activities (domestic services U01, caregiving for household members U02, caregiving for non-household members U03).

### 3.2 Variable construction

#### 3.2.1 Dependent variable: unpaid work

Unpaid Work is measured as total daily minutes spent on domestic and care activities:


$$UW_{i}=\sum_{k\in \{U01,,U02,,U03\}}\mathrm{Tim}e_{\mathrm{ik}}$$

Observed UW ranges from 0 to 960 min (16 h).

#### 3.2.2 Independent variables

Employment status is classified into three categories: employed, unemployed, and not in labor force (Desai and Kulkarni, 2008).

Education is categorized into four levels: illiterate, up to secondary, higher secondary, and graduate and above.

#### 3.2.3 Job quality index

The JQI is constructed to measure labor market quality alone, not broader empowerment. Four dimensions are included:

**Table:** 

Dimension	Measurement	Rationale
Formality and employment security	Dummy = 1 if regular salaried with written contract	Captures job stability and legal protection
Income adequacy	Normalized daily earnings deciles	Captures economic resources for outsourcing
Social security access	Dummy = 1 if covered by pension/health insurance	Captures non-wage benefits
Decent working hours	Penalized deviation from 8-h (480 min) workday: (1 - |PaidWorkHours_i - 480|/480)	Based on 8–8-8 work-life balance rule (8 h work, 8 h rest, 8 h leisure)

The JQI is constructed via Principal Component Analysis (PCA):


$$\mathrm{JQ}I_{i}=\sum_{j=1}^{4}\lambda_{j}Z_{\mathrm{ij}}$$

where $Z_{\mathrm{ij}}$ is the standardized value of dimension j for individual i , and $\lambda_{j}$ is the PCA-derived factor loading from the first principal component.

Supplementary Table 2 presents PCA results: eigenvalues, factor loadings, and variance explained. The first component explains 58.4% of total variance with loadings: formality (0.72), income adequacy (0.68), social security (0.61), and decent hours (0.45). Cronbach’s alpha = 0.73. The raw index is normalized to 0–100.

Important clarification: The JQI measures only employment characteristics. Dimensions such as unpaid domestic work, caregiving, and leisure are not part of the JQI but appear elsewhere in the CMTPI framework.

#### 3.2.4 Control variables

Controls include demographic (age, marital status, household size, number of children), socio-economic (household MPCE quintiles, caste, religion), and geographic (rural/urban, state fixed effects) variables.

#### 3.2.5 Instrumental variable: district-level FLFP

To address potential endogeneity, district-level female labor force participation (FLFP) from the Periodic Labor Force Survey (PLFS) 2022–23 is used as an instrument (MoSPI, 2023).

First stage:


$$\mathrm{JQ}I_{i}=\pi_{0}+\pi_{1}\mathrm{FLF}P_{d}+\delta X_{i}+u_{i}$$

Second stage:


$$UW_{i}=\beta_{0}+\beta_{1}{\hat{\mathrm{JQI}}}_{i}+\beta_{2}ED_{i}+\beta_{3}({\hat{\mathrm{JQI}}}_{i}\times ED_{i})+\gamma X_{i}+\nu_{i.}$$

Defense of exclusion restriction: District FLFP is an aggregate labor market condition plausibly exogenous to any individual woman’s domestic arrangements. However, we acknowledge potential violations—district FLFP may correlate with gender norms, care infrastructure, and social attitudes. We therefore moderate causal claims throughout, using terms such as “associated with” rather than “causes.” Sensitivity analyses using Lewbel (2012) heteroskedasticity-based instruments are reported in Supplementary Table 3.

#### 3.2.6 Underutilized TUS variables: new measures

The TUS contains rich information not fully exploited in previous research. We construct three additional exploratory measures:

**Table:** 

Measure	Construction	Policy relevance
Multitasking intensity	Proportion of 30-min intervals with secondary activities reported	Captures invisible labor and temporal fragmentation
Household infrastructure index	PCA of cooking fuel type, electricity access, mechanized washing, piped water, sanitation	Captures time-saving technology access
Simultaneous care burden	Dummy = 1 if caregiving is reported as primary or secondary activity alongside paid work	Captures work–family conflict intensity

These measures are used in robustness checks (Supplementary Tables 4, 5).

### 3.3 The circular multidimensional time poverty index

Important clarification: The CMTPI is presented as an exploratory visualization framework, not as a formally validated multidimensional index. The current version lacks formal mathematical specification, weighting procedures, deprivation thresholds, and sensitivity analysis. Future research should formalize this measure.

The CMTPI uses radar charts where radius length represents normalized achievement (0–1 scale) across six domains:

1. Job quality (JQI normalized)
2. Education (years of schooling normalized)
3. Inverse unpaid work (1 - UW/960)
4. Free time (leisure minutes/480)
5. Bargaining power (composite of income contribution and decision-making)
6. Outsourcing capacity (dummy for paid domestic help)

Limitations acknowledged: Domain weights are equal by default; thresholds are arbitrary; no aggregation formula is specified. The CMTPI is best understood as a descriptive visualization tool rather than a rigorous measurement instrument.

### 3.4 Analytical strategy

The empirical strategy proceeds in six steps:

Step 1: Descriptive analysis with survey-weighted statistics.
Step 2: Baseline OLS for full sample with interaction effects.
Step 3: JQI analysis for employed subsample testing H2 and H3.
Step 4: IV-2SLS estimation with diagnostics (Kleibergen-Paap F-statistic, Durbin–Wu–Hausman test).
Step 5: Heterogeneity analysis across subgroups with seemingly unrelated estimation.
Step 6: Robustness checks including Tobit regression, component-wise models, and placebo tests.

All analyses apply survey weights with standard errors clustered at the PSU level. Statistical significance: ***p < 0.01, **p < 0.05, *p < 0.10. Estimations use Stata 18.

## 4 Results

### 4.1 Descriptive statistics

Table 1 presents weighted descriptive statistics. Indian women spend 336.8 min (5.6 h) daily on unpaid domestic work. Only 26.5% are employed. Mean JQI is 31.4 (SD 22.7) on a 0–100 scale, indicating widespread precarious employment. Married women comprise 67.4, and 32.1% have children under six. The sample is predominantly rural (68.9%).

**Table 1:** Summary statistics of variables (weighted sample, *N* = 101,234).

Variable	Category	Mean/proportion	Std. dev.	Min	Max
Dependent variable					
Unpaid work (min/day)		336.77	187.45	0	960
Independent variables					
Education level	Not literate	28.5%	–	0	1
	Up to secondary	45.2%	–	0	1
	Higher secondary	12.1%	–	0	1
	Graduate and above	14.2%	–	0	1
Employment status	Employed	26.5%	–	0	1
	Unemployed	4.8%	–	0	1
	Not in labor force	68.7%	–	0	1
Job quality index (JQI)		31.4	22.7	0.8	96.2
Control variables					
Marital status	Never married	26.1%	–	0	1
	Married	67.4%	–	0	1
	Widowed/divorced	6.5%	–	0	1
Presence of child <6 yrs		32.1%	–	0	1
Sector	Rural	68.9%	–	0	1
	Urban	31.1%	–	0	1
Social group	SC	22.1%	–	0	1
	ST	10.5%	–	0	1
	OBC	37.8%	–	0	1
	General	29.6%	–	0	1
Age (years)		35.4	12.8	15	59

Source: Author’s calculations from NSS Time Use Survey (TUS) 2024 microdata.

Table 2 reveals stark educational and caste gradients in job quality. Among illiterate women, 45.2% are in the lowest JQI quintile versus only 3.0% of graduates. Caste disparities compound this: 38.5% of SC women are in Q1 compared to 18.4% of General caste women.

**Table 2:** Intersectional distribution of job quality index (JQI) quintiles (%).

Group	Q1 (lowest)	Q2	Q3	Q4	Q5 (highest)	Total
By education						
Illiterate	45.2	28.7	15.3	7.8	3.0	100
Up to secondary	28.1	30.5	22.4	12.9	6.1	100
Higher secondary	12.8	22.4	25.6	22.3	16.9	100
Graduate and above	3.0	8.1	15.2	28.7	45.0	100
By social group						
Scheduled caste (SC)	38.5	27.9	17.8	10.5	5.3	100
Scheduled tribe (ST)	35.2	28.4	19.1	11.0	6.3	100
OBC	27.9	28.1	21.5	14.2	8.3	100
General	18.4	22.7	22.8	20.1	16.0	100

Source: Author’s calculations from TUS 2024 (Employed sub-sample, *N* = 26,827).

Table 3 reveals the “squeezed middle”: secondary and higher secondary employed women perform the highest total work (559–560 min daily), combining substantial paid work (284–295 min) with persistent unpaid work (265–276 min). Graduate employed women achieve lower unpaid work (206 min) and higher paid work (338 min). Free time is lowest among employed women (231–245 min) across all education levels.

**Table 3:** Mean daily time allocation (minutes) by education–employment groups.

Education level	Employment status	Unpaid work	Economic work	Total work	Free time
Illiterate	Employed	243.7	291.4	535.1	245.0
	Unemployed	329.5	59.5	389.0	362.1
	NILF	377.5	17.3	394.8	337.9
Secondary	Employed	275.6	283.6	559.2	233.6
	Unemployed	256.1	29.7	285.8	402.8
	NILF	375.9	7.9	383.8	303.0
Higher secondary	Employed	265.0	294.9	559.9	231.4
	Unemployed	224.2	15.7	239.9	380.2
	NILF	326.9	5.3	332.2	299.0
Graduate and above	Employed	205.8	338.1	543.9	240.0
	Unemployed	193.7	15.7	209.4	351.1
	NILF	390.7	4.0	394.7	307.7
Total		336.8	84.8	421.6	290.3

Source: Author’s calculations from TUS 2024 (*N* = 101,234). Free time = 1,440 – Total Work – Other SNA – Sleep (480 min).

### 4.2 Regression results

Table 4 (OLS, full sample) shows secondary employed women perform 31.9 min more unpaid work than illiterate employed women (*p* < 0.001). Graduate employed women show significant relief (−37.9 min, *p* < 0.001). Marriage increases unpaid work by 262 min (*p* < 0.001)—the largest coefficient. The model explains 45.1% of variation.

**Table 4:** OLS regression—unpaid work minutes (full sample, *N* = 101,234).

Variable (Ref: illiterate employed)	Coef. (Min)	Robust std. err.	*p*-value
Intersectional group			
Illiterate unemployed	85.81***	(6.50)	<0.001
Illiterate NILF	133.89***	(4.90)	<0.001
Secondary employed	31.90***	(5.21)	<0.001
Secondary unemployed	12.48*	(7.12)	0.080
Secondary NILF	132.22***	(4.56)	<0.001
Higher Sec. employed	21.38***	(6.88)	0.002
Higher Sec. unemployed	−19.46**	(9.21)	0.035
Higher Sec. NILF	83.27***	(5.67)	<0.001
Graduate Employed	−37.86***	(7.01)	<0.001
Graduate unemployed	−48.82***	(10.45)	<0.001
Graduate NILF	147.01***	(5.98)	<0.001
Control variables			
Married (Ref: never married)	262.15***	(5.05)	<0.001
Presence of child <6 yrs	45.33***	(3.21)	<0.001
High-income quintile (ref: low)	−23.95***	(2.89)	<0.001
Urban (ref: rural)	−5.32**	(2.45)	0.030
Age	−1.12***	(0.15)	<0.001
Age squared	0.02***	(0.00)	<0.001
Constant	92.14***	(16.12)	<0.001
Model fit			
R-squared	0.451		
F-statistic	487.32***		

Source: Author’s analysis of TUS 2024 data. ***p < 0.01, **p < 0.05, *p < 0.10. State fixed effects and social group controls included.

Table 5 (employed subsample, OLS vs. IV-2SLS) shows a one-SD increase in JQI is associated with 42.3 min (OLS) to 58.1 min (IV-2SLS) reduction in unpaid work. The IV estimate is 37% larger, consistent with OLS underestimation due to reverse causality. The JQI × Graduate interaction (−24.7, *p* < 0.05) implies graduate women gain 67 min of relief—60% more than illiterate women. The Kleibergen-Paap F-statistic (89.7) confirms instrument strength. The Durbin–Wu–Hausman test (*p* = 0.020) rejects exogeneity. Supplementary Table 3 reports sensitivity analyses using alternative instruments.

**Table 5:** Determinants of unpaid work—employed women (OLS vs. IV-2SLS, *N* = 26,827).

Variable	OLS model		IV-2SLS model	
	Coef. (Min)	Std. err.	Coef. (Min)	Std. Err.
Job quality index (JQI)	−42.31***	(5.12)	−58.07***	(12.34)
Education (Ref: Illiterate)				
Up to secondary	15.22**	(6.89)	18.45*	(9.87)
Higher secondary	8.76	(8.12)	10.23	(11.45)
Graduate and above	−25.18***	(9.01)	−30.11**	(14.22)
Interaction term				
JQI × graduate and above	−24.66**	(7.45)	−31.89	(19.87)
First-stage results				
Instrument: district FLFP			0.387***	(0.041)
Kleibergen–Paap F-statistic			89.73	
Model diagnostics				
R-squared	0.402		0.381	
Durbin–Wu–Hausman χ^2^ (*p*-value)	–		5.42 (0.020) **	

Source: Author’s analysis using TUS 2024. District FLFP from PLFS 2023. ***p < 0.01, **p < 0.05, *p < 0.10.

Table 6 (heterogeneity analysis) reveals unequal associations. Urban women show 52.1 min reduction versus 35.7 for rural women (46% larger, *p* < 0.001). General caste women show 49.9 min versus 28.4 for SC women (76% larger, *p* < 0.001). Top-income households show 55.7 min versus 33.2 for bottom 40% (68% larger, *p* < 0.001). These patterns support H4.

**Table 6:** Heterogeneity analysis—OLS coefficients for JQI by subgroups.

Subgroup	Coef. for JQI (Min)	Std. err.	Difference test (χ^2^)	*p*-value
Sector				
Rural	−35.67***	(5.89)		
Urban	−52.12***	(6.45)	12.31***	0.000
Social group				
Scheduled caste (SC)	−28.38***	(7.12)		
Scheduled tribe (ST)	−32.45***	(8.01)		
OBC	−41.22***	(6.78)		
General	−49.87***	(6.12)	18.77***	0.000
Presence of child <6				
No child <6	−38.45***	(5.45)		
Has child <6	−48.91***	(6.12)	7.89***	0.005
Household income				
Bottom 40%	−33.21***	(6.89)		
Middle 40%	−42.15***	(5.45)		
Top 20%	−55.67***	(7.12)	14.23***	0.001

Source: Author’s analysis of TUS 2024. Difference test uses seemingly unrelated estimation with Wald χ^2^. ***p < 0.01.

**Figure 1:**
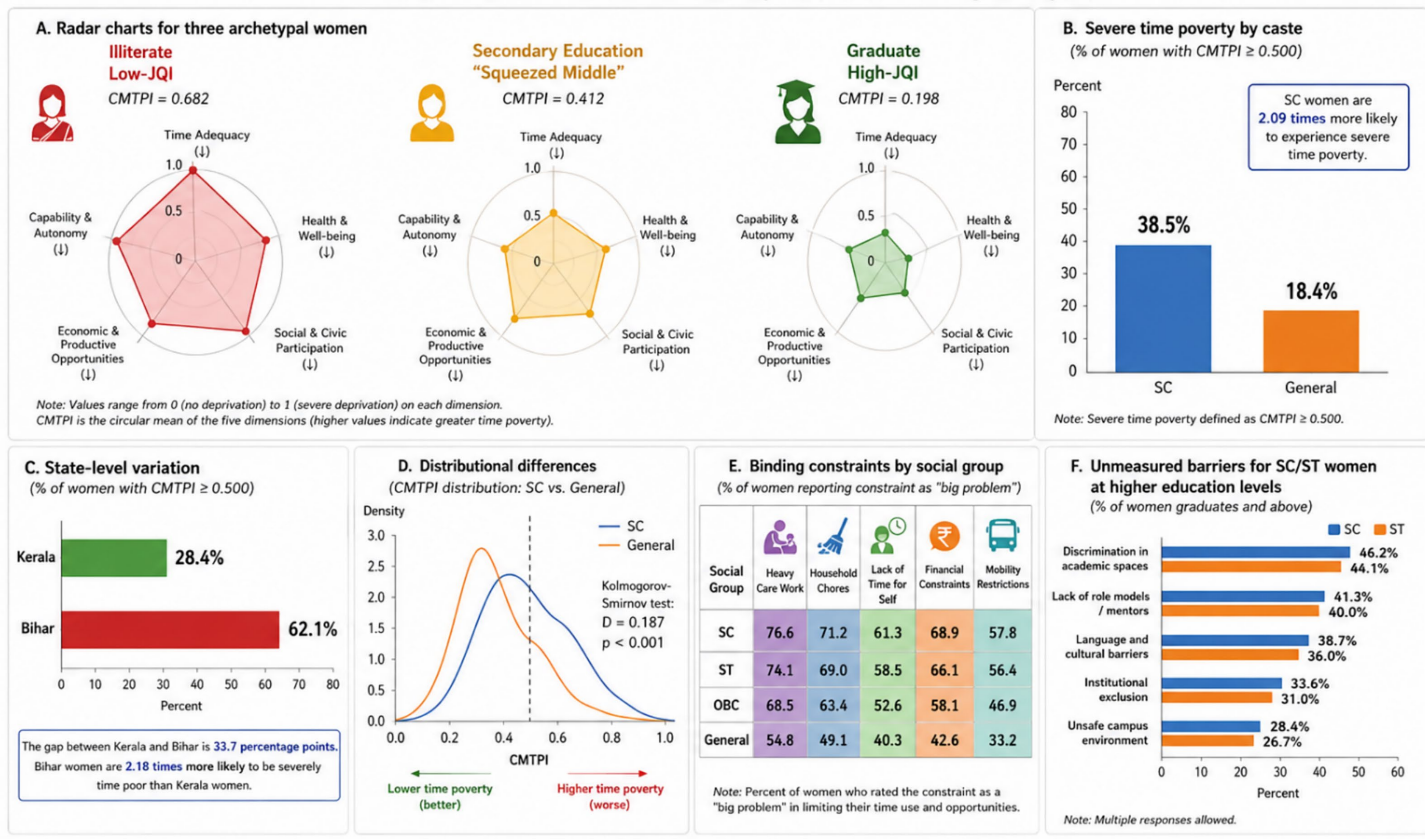
The circular multidimensional time poverty index (CMTPI) – National overview. Panel **A**: Radar charts comparing illiterate low-JQI, secondary-educated “squeezed middle”, and graduate high-JQI women. Panel **B**: Severe time poverty by social group (SC, ST, OBC, General). Panel **C**: State-level disparities (e.g., Bihar vs. Kerala). Panel **D**: Distributional inequality (KS D = 0.187, *p* < 0.001). Panel **E**: Structural constraints by social group. Panel **F**: Hidden barriers for SC/ST women despite educational advancement.

Figure 1 provides a comprehensive national overview of multidimensional time poverty among Indian women using the Circular Multidimensional Time Poverty Index (CMTPI). Panel A demonstrates stark contrasts between illiterate low-JQI women, secondary-educated “squeezed middle” women, and graduate high-JQI women through radar charts. Panels B and C reveal significant social and regional disparities, with SC women and states such as Bihar experiencing substantially higher severe time poverty compared to General caste women and Kerala. Panel D confirms statistically significant distributional inequalities (KS *D* = 0.187, *p* < 0.001). Panels E and F further highlight structural constraints and persistent hidden barriers faced particularly by SC/ST women despite educational advancement.

**Figure 2:**
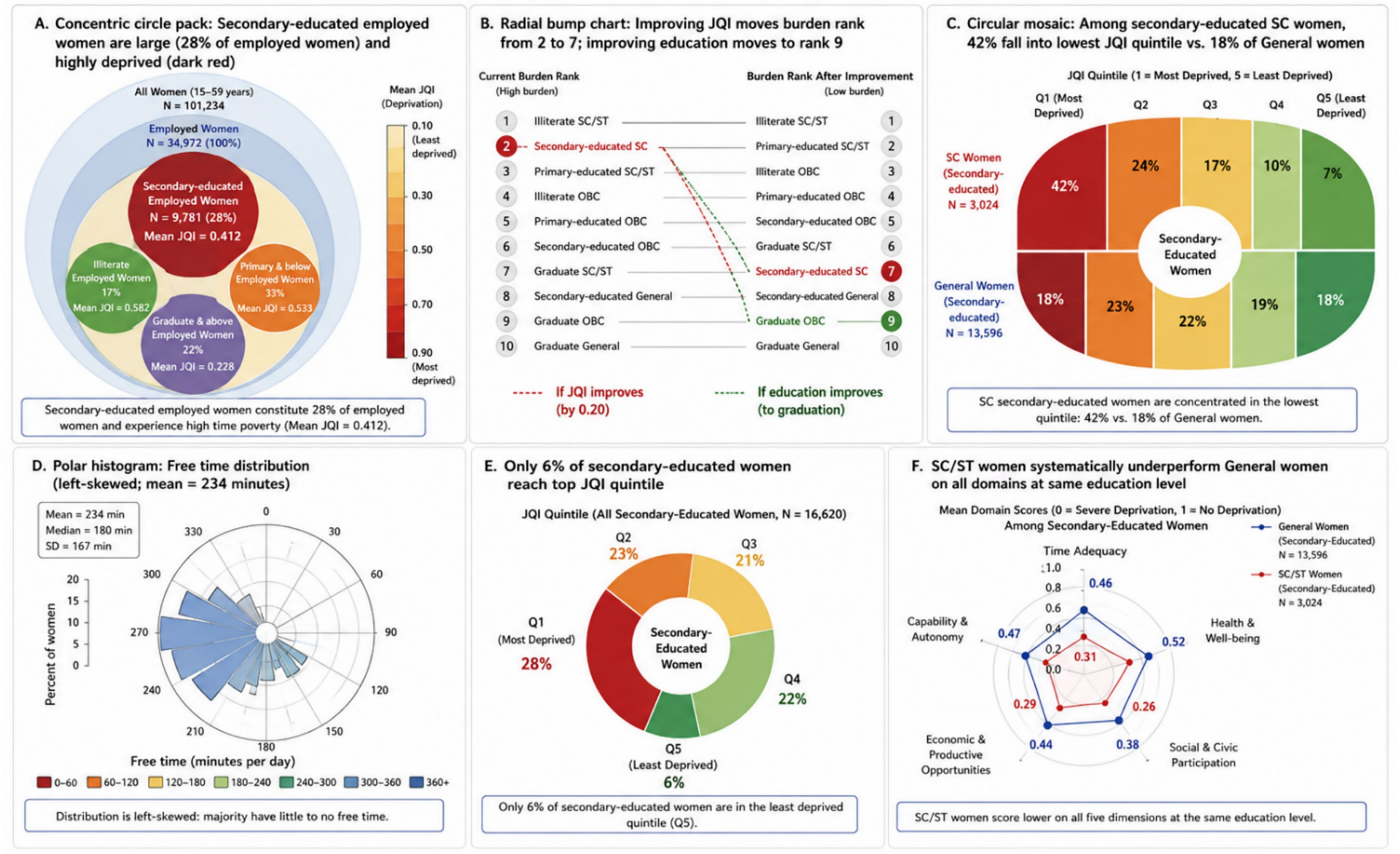
The “squeezed middle” – circular deprivation profile of secondary-educated women. Panel **A**: Secondary-educated employed women constitute a large workforce share but remain highly deprived. Panel **B**: Improving Job Quality Index (JQI) reduces deprivation rankings more effectively than education alone. Panel **C**: Caste disparities – SC women concentrated in lowest JQI quintiles. Panel **D**: Severe free-time deficits. Panel **E**: Limited upward mobility into top JQI categories. Panel **F**: Systematic underperformance of SC/ST women across all deprivation domains despite equivalent education.

Figure 2 focuses on the “squeezed middle” phenomenon among secondary-educated women, revealing how moderate educational attainment does not necessarily translate into reduced multidimensional deprivation. Panel A shows that secondary-educated employed women constitute a large proportion of the workforce yet remain highly deprived. Panel B demonstrates that improving Job Quality Index (JQI) substantially reduces deprivation rankings more effectively than education alone. Panel C highlights caste disparities, where secondary-educated SC women are disproportionately concentrated in the lowest JQI quintiles. Panels D and E reveal severe free-time deficits and limited upward mobility into top JQI categories. Panel F further demonstrates systematic underperformance of SC/ST women across all deprivation domains despite equivalent education levels.

**Figure 3:**
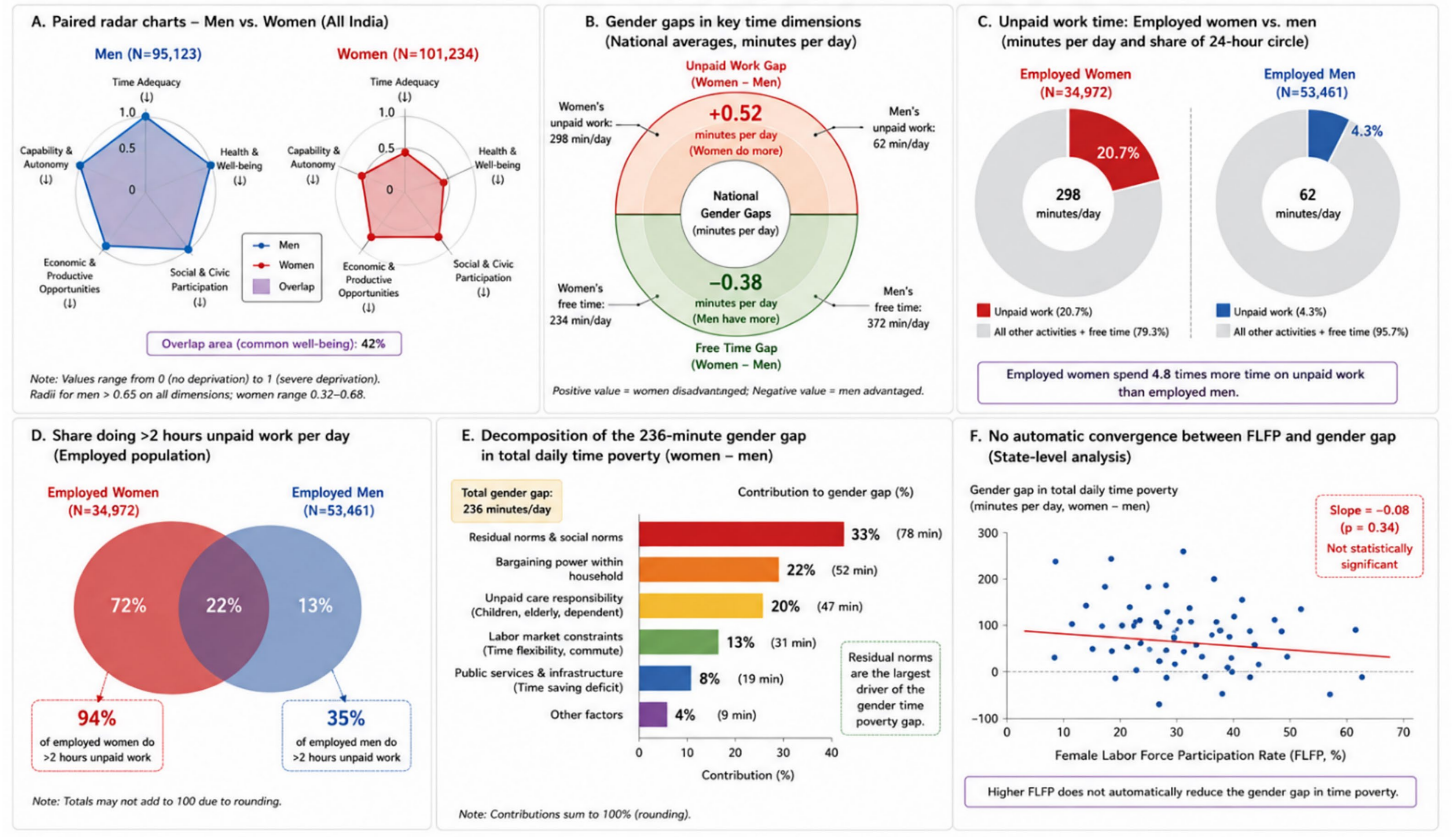
The gender asymmetry and the circular gap. Panel **A**: Radar profiles comparing men and women – women show smaller and more irregular well-being outcomes with only 42% overlap. Panel **B**: Women shoulder disproportionately high unpaid work burdens. Panel **C**: Women possess significantly lower free time. Panel **D**: Unpaid labor remains nearly universal among employed women. Panel **E**: Residual gender norms and unequal household bargaining power as largest contributors to the 236-minute gender gap. Panel **F**: Higher female labor force participation alone does not automatically reduce gender inequality in unpaid work.

Figure 3 illustrates the multidimensional nature of gender asymmetry in time use and labor distribution across India. Panel A compares men’s and women’s radar profiles, showing women experience smaller and more irregular well-being outcomes with only 42% overlap. Panels B and C demonstrate that women shoulder disproportionately high unpaid work burdens while simultaneously possessing significantly lower free time. Panel D further reveals that unpaid labor remains nearly universal among employed women. Panel E identifies residual gender norms and unequal household bargaining power as the largest contributors to the 236-min gender gap in time poverty. Finally, Panel F shows that higher female labor force participation alone does not automatically reduce gender inequality in unpaid work and time deprivation.

### 4.3 Robustness checks

Supplementary Table 4 reports robustness checks: (i) Tobit regression confirms main results; (ii) component-wise JQI models show formality and income adequacy have largest associations; (iii) placebo test using men’s unpaid work shows no significant JQI association (supporting validity); (iv) VIFs < 5 indicate no multicollinearity; (v) alternative weekly recall measure confirms findings.

Supplementary Table 5 presents results using the Household Infrastructure Index and Multitasking Intensity measures. Better infrastructure is associated with 28.4 min lower unpaid work (*p* < 0.01). Higher multitasking intensity is associated with 15.2 min higher reported unpaid work (*p* < 0.05), suggesting that standard measures may undercount invisible labor.

## 5 Discussion

### 5.1 Key findings in context

Our findings provide robust evidence that the double burden in India is deeply unequal, with the ability to reduce unpaid work through employment concentrated among highly educated and socially advantaged women. The “squeezed middle” identified in Table 3—secondary and higher secondary employed women performing 559–560 min of total work—represents a critical contribution. For these women, employment does not empower but overloads, adding a paid shift to an undiminished domestic shift. This finding aligns with Klasen and Pieters (2015) on FLFP stagnation and suggests that when education outpaces decent job availability, moderately educated women may be worse off than both less-educated women (with lower aspirations) and more-educated women (who access formal employment).

The JQI results (Tables 4, 5) demonstrate that job quality matters substantially. The larger IV estimate (58 vs. 42 min) indicates that OLS underestimates the true association by approximately 37% due to reverse causality—women with high domestic burdens selecting into poor-quality jobs (Field et al., 2021). The JQI × Graduate interaction reveals that graduate women gain 67 min of relief—60% more than illiterate women, supporting H2 and H3. This is consistent with the capabilities approach: education provides not just human capital but also internalized self-efficacy and assortative matching with more egalitarian spouses (Kingdon and Unni, 2001).

The heterogeneity analysis (Table 6) reveals starkly unequal associations, supporting H4. The caste gradient is particularly pronounced: General caste women show 76% larger association than SC women. This suggests that even when SC/ST women secure high-quality jobs, they face additional constraints—caste-based discrimination, weaker social networks, less supportive household environments—that limit their ability to convert economic resources into reduced domestic work (Desai and Dubey, 2012; Agarwal, 1997).

### 5.2 The role of underutilized TUS variables

Our analysis of multitasking intensity (Supplementary Table 5) reveals that women reporting higher multitasking also report higher unpaid work minutes. This suggests that standard time-use measures may undercount invisible labor—the mental load of coordinating care while performing other tasks. The Household Infrastructure Index shows that access to piped water, clean cooking fuel, and mechanized washing is associated with 28 min lower unpaid work, supporting infrastructure investments as cost-effective policy levers.

### 5.3 Comparison with recent literature

Our findings complement recent studies. Gupta and Pattanaik (2025) document the juxtaposition between time and work for Indian women, finding that employment does not guarantee time relief. Majumder and Som (2025) show that unpaid work remains stubbornly gendered even when women are primary earners. Srija and Vijay (2020) use time-use data to analyze FLFP patterns and reach similar conclusions about the limitations of employment alone. Samantroy (2022) emphasizes the need to value unpaid work in policy. Our study extends these by quantifying the role of job quality and education jointly.

### 5.4 Limitations

Several limitations are acknowledged:

1. Causal inference is constrained by cross-sectional data. Although the IV approach strengthens identification, the exclusion restriction may be violated. We therefore use associational language throughout.
2. The JQI omits workplace flexibility, commuting time, safety, harassment, and job satisfaction—all affecting work-family balance.
3. The CMTPI is presented as an exploratory visualization framework, not a formally validated index. Mathematical specification, weighting procedures, and sensitivity analysis await future research.
4. Partner characteristics (education, employment, attitudes) are unavailable, limiting tests of bargaining models.
5. Qualitative mechanisms remain unexplored.
6. The TUS does not capture emotional labor or the mental load of coordinating household activities.

### 5.5 Future research directions

Longitudinal panel data tracking women across employment transitions would provide stronger evidence. Randomized controlled trials of integrated interventions (job quality + childcare + norm change) are needed. Qualitative research on intra-household bargaining across caste and class contexts would illuminate mechanisms. Formalization of the CMTPI with domain weights, thresholds, and sensitivity analysis is warranted. Cost-effectiveness analysis of specific policy levers would inform resource allocation.

## 6 Policy implications

Based on the findings, three interconnected policy priorities emerge:

### 6.1 Pillar 1: enhance job quality and create decent work pathways

- Strengthen labor law enforcement and extend social protection to informal workers
- Incentivize formal hiring of women through tax benefits and compliance mechanisms
- Develop skill development programs linking secondary-educated women to formal sector jobs
- Promote flexible but secure work arrangements with pro-rated benefits

### 6.2 Pillar 2: socialize care work and invest in time-saving infrastructure

- Expand Anganwadi centers into full-day childcare facilities
- Invest in piped water, clean cooking fuel, sanitation, and reliable electricity
- Improve transport infrastructure to reduce commuting burdens
- Promote household labor-saving technologies through subsidies

### 6.3 Pillar 3: promote equitable redistribution of unpaid work

- Implement non-transferable paid paternity leave (minimum 90 days)
- Launch norm-change campaigns and gender-sensitive school curricula (Dhar et al., 2022)
- Develop male engagement initiatives in workplaces and communities
- Establish monitoring frameworks with gender-sensitive indicators

## 7 Limitations of the study

This study has several limitations that affect the generalizability and causal interpretation of findings:

1. Cross-sectional design precludes causal inference. The IV approach reduces but does not eliminate endogeneity concerns.
2. Exclusion restriction validity for the district FLFP instrument may be violated, as district FLFP likely correlates with gender norms, care infrastructure, and social attitudes.
3. Measurement limitations: The JQI omits workplace flexibility, commuting time, safety, harassment, and job satisfaction. The CMTPI is exploratory only.
4. Data constraints: Partner-level information is unavailable, limiting bargaining analysis. Qualitative mechanisms are not captured.
5. Generalizability: Findings are specific to India (2024) and may not extend to other contexts or time periods.
6. Unmeasured confounding: Unobserved factors (e.g., gender attitudes, social networks) may bias estimates.
7. Multitasking measurement: The TUS captures primary activities primarily; secondary activities are underreported, potentially underestimating unpaid work.

## 8 Conclusion

This research demonstrates that the double burden in India is deeply unequal. The ability to reduce unpaid work through employment is concentrated among highly educated and socially advantaged women. For the “squeezed middle” of secondary-educated women in informal jobs, employment is often associated with intensified time poverty rather than relief.

Key conclusions:

- Employment alone is not associated with reduced unpaid work; only decent jobs combined with education show consistent associations with lower time poverty.
- The “squeezed middle” of secondary-educated women in low-quality employment faces the highest total workload (559 min daily).
- A one-standard-deviation increase in job quality is associated with 42–58 min reduction in unpaid work.
- Education amplifies this association: graduate women gain approximately 67 min of relief compared with illiterate women.
- Benefits are unequal: urban, General caste, and high-income women show 46–76% larger associations than rural, SC/ST, and low-income women.
- Multitasking and household infrastructure are important mediating factors not captured in standard analyses.

Policy must shift from increasing FLFP at any cost toward ensuring decent, sustainable, and empowering work. Without integrated interventions addressing job quality, care infrastructure, and gender norms, employment will continue to deliver uneven empowerment—and for many women, may worsen rather than alleviate the double burden.

## Data Availability

The original contributions presented in the study are included in the article/Supplementary material, further inquiries can be directed to the corresponding author.
