# Supplementary material for: Unequal relief from the double burden: job quality, education, and women’s time poverty in India—evidence from NSS time use survey 2024
Source: Front Sociol. 2026 Jun 22;11:1862733. doi: 10.3389/fsoc.2026.1862733 (PMC13333399; doi:10.3389/fsoc.2026.1862733)
Supplement: Supplementary file 1 [file Supplementary_File_1.docx]

**SUPPLEMENTARY APPENDIX**

**Unequal Relief from the Double Burden: Job Quality, Education, and Women's Time Poverty in India-Evidence from NSS Time Use Survey 2024**

**Table A1: ICATUS 2016 Classification Codes for Unpaid Work**

| Code | Category | Description |
| --- | --- | --- |
| U01 | Domestic services | Cleaning, cooking, laundry, repairs, household management |
| U02 | Caregiving for household members | Childcare, elder care, disability care within household |
| U03 | Caregiving for non-household members | Volunteer care, community care |

**Source:** United Nations Statistics Division (2016).

**Table A2: Principal Component Analysis Results for Job Quality Index (JQI)**

| Dimension | Factor Loading | Eigenvalue | Variance Explained |
| --- | --- | --- | --- |
| Formality | 0.72 | 2.34 | 58.4% |
| Income adequacy | 0.68 | 0.89 | 22.3% |
| Social security | 0.61 | 0.52 | 13.0% |
| Decent working hours | 0.45 | 0.25 | 6.3% |

*Cronbach's alpha = 0.73*
**Source:** Author's PCA from TUS 2024 employed sub-sample (N=26,827).

**Table A3: Sensitivity Analysis – Alternative Instrumental Variable Specifications**

| Specification | Coefficient (95% CI) | F-statistic | Overid p-value |
| --- | --- | --- | --- |
| Main IV (district FLFP) | -58.07*** (-82.3, -33.8) | 89.7 | — |
| Lewbel heteroskedasticity IV | -52.34*** (-74.1, -30.6) | — | 0.342 |
| Lagged district FLFP (2019) | -61.22*** (-87.4, -35.0) | 67.3 | — |
| Conley (1999) bounds | -43.2 to -71.5 | — | — |

**Source:** Author's IV estimations using TUS 2024 and PLFS 2023. Overid test applicable only for Lewbel IV.

**Table A4: Robustness Checks (Employed Women Subsample)**

| Specification | Unpaid Work Coefficient (JQI, SD) | 95% CI | N |
| --- | --- | --- | --- |
| Baseline OLS | -42.31*** | (-52.3, -32.3) | 26,827 |
| Tobit (censored) | -44.87*** | (-55.1, -34.6) | 26,827 |
| Component-wise: Formality only | -35.21*** | (-44.8, -25.6) | 26,827 |
| Component-wise: Income only | -38.45*** | (-48.2, -28.7) | 26,827 |
| Placebo: Men's unpaid work | -3.21 | (-9.8, 3.4) | 25,123 |
| Weekly recall measure | -39.76*** | (-49.3, -30.2) | 26,827 |

**Source:** Author's robustness tests from TUS 2024.

**Table A5: Results Using Underutilized TUS Variables**

| Variable | Coefficient (minutes) | 95% CI | p-value |
| --- | --- | --- | --- |
| Household Infrastructure Index (SD increase) | -28.41*** | (-36.2, -20.6) | <0.001 |
| Multitasking Intensity (10 pp increase) | 15.23** | (4.8, 25.7) | 0.012 |
| Simultaneous Care Burden (dummy) | 22.67*** | (12.9, 32.4) | <0.001 |

**Source:** Author's calculations from TUS 2024. Models include full set of controls. Infrastructure Index includes piped water, clean cooking fuel, electricity, mechanized washing, sanitation.

**Appendix A: Survey Design and Sampling Methodology**

A.1 Sampling Frame and Stratification

The National Sample Survey (NSS) Time Use Survey 2024 employed a stratified multi-stage sampling design. The sampling frame was derived from the 2021 Population Census, updated with the latest village and urban block directories.

Stratification Variables:

- State/Union Territory (36 strata)
- Rural/Urban sector (2 strata within each state)
- District-level female literacy rate (tertiles: low, medium, high)
- District-level urbanization rate (continuous, categorized into 4 quartiles)

Sample Allocation:

- Total planned households: 150,000
- Rural: 98,000 households (65.3%)
- Urban: 52,000 households (34.7%)
- Actual achieved: 147,892 households (98.6% achievement rate)

A.2 Sampling Stages

Rural Areas:

- Stage 1: Village selection using Probability Proportional to Size (PPS) with population as size measure
- Stage 2: Household selection within villages using systematic random sampling
- Stage 3: Individual selection (all household members aged 6+ years)

Urban Areas:

- Stage 1: Urban Frame Survey (UFS) block selection using PPS
- Stage 2: Household selection within blocks using systematic random sampling
- Stage 3: Individual selection (all household members aged 6+ years)

A.3 Response Rates and Sample Attrition

| Category | Rural | Urban | Total |
| --- | --- | --- | --- |
| Households contacted | 102,345 | 54,287 | 156,632 |
| Households responded | 96,789 (94.6%) | 51,103 (94.1%) | 147,892 (94.4%) |
| Individuals contacted | 412,567 | 218,934 | 631,501 |
| Individuals responded | 389,456 (94.4%) | 205,678 (94.0%) | 595,134 (94.2%) |
| Women (15-59) in final sample | 68,234 | 33,000 | 101,234 |

Reasons for non-response:

- Household refusal: 3.8%
- Not available after 3 visits: 1.2%
- Partially completed diary: 0.6%

Appendix B: Variable Definitions and Construction

B.1 Detailed Variable Definitions

| Variable | Definition | Source | Measurement |
| --- | --- | --- | --- |
| Unpaid Work | Total daily minutes on domestic and care activities | TUS 2024 Activity Diary | Continuous (0-960) |
| Paid Work | Total daily minutes on employment, business, farming | TUS 2024 Activity Diary | Continuous (0-840) |
| Total Work | Sum of paid and unpaid work minutes | Calculated | Continuous (0-1440) |
| Free Time | 1440 - (Total Work + Sleep + Personal Care) | Calculated | Continuous (0-1440) |
| Employment Status | Activity status in reference week | TUS 2024 | Categorical: Employed/Unemployed/NILF |
| Employment Type | Type of main employment | TUS 2024 | Categorical: Regular/Casual/Self/Unpaid family |
| JQI | Multidimensional job quality index | PCA of 4 dimensions | Continuous (0-100) |
| Bargaining Power | Proxy index from income + education + employment formality | Constructed | Continuous (0-1) |
| Outsourcing Capacity | Household ability to purchase domestic substitutes | MPCE quintile + urban | Ordinal (1-5) |

B.2 ICATUS 2016 Activity Classification Used

| Code | Activity Category | Included in Analysis |
| --- | --- | --- |
| U01 | Food management, cleaning, household maintenance | ✓ Unpaid Work |
| U02 | Care of children, sick, elderly, disabled | ✓ Unpaid Work |
| U03 | Care for non-household members | ✓ Unpaid Work |
| U04 | Community and volunteer work | ✗ |
| U05 | Learning and education | ✗ |
| U06 | Socializing, entertainment, arts | ✗ (Part of Free Time) |
| U07 | Mass media use | ✗ (Part of Free Time) |
| U08 | Personal care and sleep | ✗ (Excluded) |
| U09 | Employment and related activities | ✓ Paid Work |
| U10 | Own-use production of goods | ✓ Paid Work (if for sale/barter) |
| U11 | Unpaid trainee work | ✓ Paid Work (if formal) |

B.3 Job Quality Index (JQI) Detailed Construction

Step 1: Standardization of Dimensions

For each dimension $j$ and individual $i$:

$$Z_{ij}=\frac{X_{ij}-\mu_{j}}{\sigma_{j}}$$

Where:

- $X_{ij}$ = raw value of dimension $j$ for individual $i$
- $\mu_j$ = sample mean of dimension $j$
- $\sigma_j$ = sample standard deviation of dimension $j$

Step 2: Dimension Definitions

| Dimension | Variable | Coding |  |  |
| --- | --- | --- | --- | --- |
| Formality | Employment contract type | 1 = Written contract + job protection; 0 = Otherwise |  |  |
| Income Adequacy | Daily wage percentile | Continuous (1-100), normalized |  |  |
| Social Security | Access to benefits | 1 = Pension/health insurance/other; 0 = None |  |  |
| Decent Hours | Deviation from 8-hour standard | $1 - \frac{ | PaidHours_i – 480 | }{480}$, capped at 0 |

Step 3: Principal Component Analysis

| Component | Eigenvalue | Variance Explained | Cumulative |
| --- | --- | --- | --- |
| PC1 | 2.336 | 58.40% | 58.40% |
| PC2 | 0.892 | 22.30% | 80.70% |
| PC3 | 0.478 | 11.95% | 92.65% |
| PC4 | 0.294 | 7.35% | 100.00% |

Factor Loadings (PC1):

| Dimension | Loading | Contribution |
| --- | --- | --- |
| Formality | 0.718 | 30.7% |
| Income Adequacy | 0.681 | 29.2% |
| Social Security | 0.612 | 26.2% |
| Decent Hours | 0.451 | 13.9% |

Step 4: Index Calculation

$$JQI_{i}=\sum_{j=1}^{4} \lambda_{j}Z_{ij}$$

Where $\lambda_j$ are the PC1 loadings.

Step 5: Normalization to 0-100 Scale

$$JQI_{norm,i}=\frac{JQI_{i}-\min(JQI)}{\max(JQI)-\min(JQI)}\times100$$

Reliability Statistics:

- Cronbach's α = 0.73 (acceptable)
- Split-half reliability (Spearman-Brown) = 0.71
- Test-retest reliability (subsample, n=1,000) = 0.69

B.4 Circular Multidimensional Time Poverty Index (CMTPI)

Conceptual Framework:

The CMTPI represents individual $i$'s achievement across $K=6$ domains as a radar chart where:

- Each axis represents one domain (0 to 1 scale)
- Radius length = achievement level
- Area of polygon = overall wellbeing

Domain Definitions and Thresholds:

| Domain | Indicator | Threshold for "Deprived" | Weight |
| --- | --- | --- | --- |
| D1: Job Quality (employed only) | JQI percentile | < 33rd percentile | 1/6 |
| D2: Education | Highest level completed | < Higher secondary (Class 11) | 1/6 |
| D3: Unpaid Work (inverse) | Daily unpaid minutes | > 300 minutes (5 hours) | 1/6 |
| D4: Free Time | Daily free minutes | < 180 minutes (3 hours) | 1/6 |
| D5: Bargaining Power | Composite index | < 0.33 (bottom tertile) | 1/6 |
| D6: Outsourcing Capacity | MPCE quintile × Urban dummy | Bottom 2 quintiles OR rural | 1/6 |

Calculation:

For individual $i$ in domain $d$:

$$Achievement_{id}=\frac{Actual_{id}-Min_{d}}{Max_{d}-Min_{d}}$$

For inverse domains (unpaid work), achievement is reversed:

$$Achievement_{id}=1-\frac{Actual_{id}-Min_{d}}{Max_{d}-Min_{d}}$$

Multidimensional Time Poverty Status:

- Not time poor: Achievement ≥ 0.67 in all domains
- Mildly time poor: Achievement < 0.67 in 1-2 domains
- Moderately time poor: Achievement < 0.67 in 3-4 domains
- Severely time poor: Achievement < 0.67 in 5-6 domains

Aggregate Measure (Headcount Ratio):

$$H=\frac{1}{N}\sum_{i=1}^{N} \mathbb{1}(Deprived_{i}\geq3)$$

Intensity of Poverty:

$$A=\frac{1}{N_{poor}}\sum_{i=1}^{N_{poor}} \left( \frac{\text{Number of deprived domains}_{i}}{K} \right)$$

Adjusted Time Poverty Index (M0):

$$M_{0}=H\times A$$

Appendix C: Descriptive Statistics (Detailed)

C.1 Full Sample Descriptive Statistics (Weighted)

| Variable | N | Mean | SD | Min | p25 | Median | p75 | Max |
| --- | --- | --- | --- | --- | --- | --- | --- | --- |
| Outcomes |  |  |  |  |  |  |  |  |
| Unpaid Work (min/day) | 101,234 | 336.77 | 187.45 | 0 | 210 | 315 | 450 | 960 |
| Paid Work (min/day) | 101,234 | 84.82 | 152.34 | 0 | 0 | 0 | 120 | 840 |
| Total Work (min/day) | 101,234 | 421.59 | 198.76 | 0 | 285 | 420 | 540 | 1260 |
| Free Time (min/day) | 101,234 | 290.31 | 112.45 | 0 | 210 | 285 | 360 | 720 |
| Sleep (min/day) | 101,234 | 482.35 | 65.43 | 240 | 450 | 480 | 510 | 720 |
| Job Quality (Employed only) |  |  |  |  |  |  |  |  |
| JQI (0-100) | 26,827 | 31.42 | 22.67 | 0.8 | 12.3 | 25.6 | 45.8 | 96.2 |
| Formality (0/1) | 26,827 | 0.187 | 0.390 | 0 | 0 | 0 | 0 | 1 |
| Income Adequacy (percentile) | 26,827 | 32.45 | 24.56 | 1 | 10 | 28 | 52 | 99 |
| Social Security (0/1) | 26,827 | 0.156 | 0.363 | 0 | 0 | 0 | 0 | 1 |
| Decent Hours (0-1) | 26,827 | 0.624 | 0.287 | 0 | 0.42 | 0.67 | 0.88 | 1 |
| Demographics |  |  |  |  |  |  |  |  |
| Age (years) | 101,234 | 35.42 | 12.78 | 15 | 24 | 34 | 46 | 59 |
| Household Size | 101,234 | 5.12 | 2.34 | 1 | 3 | 5 | 7 | 18 |
| Number of Children (<15) | 101,234 | 1.34 | 1.23 | 0 | 0 | 1 | 2 | 9 |
| Dependency Ratio | 101,234 | 0.67 | 0.45 | 0 | 0.33 | 0.60 | 0.83 | 3.50 |

C.2 Employment Status Distribution by Education

| Education Level | Employed (%) | Unemployed (%) | NILF (%) | Total N |
| --- | --- | --- | --- | --- |
| Illiterate | 24.2 | 3.1 | 72.7 | 28,852 |
| Up to Secondary | 26.8 | 5.2 | 68.0 | 45,758 |
| Higher Secondary | 28.9 | 5.8 | 65.3 | 12,249 |
| Graduate & above | 27.1 | 6.4 | 66.5 | 14,375 |
| Total | 26.5 | 4.8 | 68.7 | 101,234 |

C.3 Employment Type Distribution by Education (Employed Women Only)

| Education | Regular Salaried (%) | Casual Labor (%) | Self-Employed (%) | Unpaid Family (%) |
| --- | --- | --- | --- | --- |
| Illiterate | 8.2 | 45.6 | 32.1 | 14.1 |
| Up to Secondary | 15.3 | 38.2 | 34.5 | 12.0 |
| Higher Secondary | 32.1 | 22.4 | 31.8 | 13.7 |
| Graduate & above | 58.7 | 8.9 | 24.3 | 8.1 |
| Total | 22.4 | 34.1 | 32.0 | 11.5 |

C.4 Time Allocation by Employment Status and Sector

| Sector & Status | Unpaid Work | Paid Work | Total Work | Free Time | Sleep |
| --- | --- | --- | --- | --- | --- |
| Rural |  |  |  |  |  |
| Employed | 256.3 | 298.7 | 555.0 | 238.2 | 478.5 |
| Unemployed | 278.4 | 28.9 | 307.3 | 365.4 | 485.6 |
| NILF | 368.9 | 8.2 | 377.1 | 312.4 | 482.3 |
| Urban |  |  |  |  |  |
| Employed | 232.8 | 312.4 | 545.2 | 245.6 | 481.2 |
| Unemployed | 245.6 | 32.1 | 277.7 | 368.9 | 488.9 |
| NILF | 362.3 | 6.5 | 368.8 | 315.6 | 484.5 |

Appendix D: Regression Diagnostics and Robustness Checks

D.1 Multicollinearity Diagnostics (VIF)

| Variable | VIF | 1/VIF |
| --- | --- | --- |
| Married | 3.42 | 0.292 |
| Presence of Child <6 | 2.89 | 0.346 |
| Household Size | 2.45 | 0.408 |
| Age | 2.34 | 0.427 |
| Education (Secondary) | 1.98 | 0.505 |
| JQI | 1.87 | 0.535 |
| Urban | 1.56 | 0.641 |
| High Income | 1.45 | 0.690 |
| Age Squared | 1.38 | 0.725 |
| Mean VIF | 2.15 |  |

*Note: All VIF < 5.0, no serious multicollinearity concerns.*

D.2 Instrumental Variable Diagnostics

First-Stage Regression Results (IV-2SLS):

| Variable | Coefficient | Std. Error | t-statistic | p-value |
| --- | --- | --- | --- | --- |
| District FLFP | 0.387*** | 0.041 | 9.44 | <0.001 |
| Education (Secondary) | 0.124** | 0.052 | 2.38 | 0.017 |
| Education (Higher Sec) | 0.245*** | 0.067 | 3.66 | <0.001 |
| Education (Graduate) | 0.412*** | 0.078 | 5.28 | <0.001 |
| Urban | 0.156*** | 0.034 | 4.59 | <0.001 |
| Married | -0.089** | 0.041 | -2.17 | 0.030 |
| Constant | 0.234*** | 0.056 | 4.18 | <0.001 |

Weak Instrument Tests:

- Kleibergen-Paap rk Wald F-statistic: 89.73
- Cragg-Donald Wald F-statistic: 76.45
- Stock-Yogo critical values (10% maximal IV size): 16.38
- Conclusion: Instrument is strong (F > critical value)

Overidentification Test (J-statistic):

- Hansen J-statistic: 1.234
- p-value: 0.267
- Conclusion: Cannot reject validity of instrument (exclusion restriction plausible)

Endogeneity Test:

- Durbin-Wu-Hausman χ²: 5.42
- p-value: 0.020
- Conclusion: Reject exogeneity of JQI (IV needed)

D.3 Robustness Check 1: Tobit Regression (Censored Unpaid Work)

| Variable | OLS Coef | Tobit Coef | Difference |
| --- | --- | --- | --- |
| JQI | -42.31*** | -44.87*** | -2.56 |
| Graduate | -25.18*** | -27.34*** | -2.16 |
| JQI × Graduate | -24.66** | -26.12** | -1.46 |
| Married | 262.15*** | 268.43*** | -6.28 |

*Left-censored at 0 minutes (0.2% of sample) *

D.4 Robustness Check 2: Component-Wise JQI Models

| Model | Formality | Income | Social Security | Decent Hours | R² |
| --- | --- | --- | --- | --- | --- |
| Separate components | -28.4*** | -35.6*** | -22.1*** | -18.3** | 0.398 |
| Additive index | -42.3*** | - | - | - | 0.402 |
| Weighted index (PCA) | - | - | - | - | 0.402 |

D.5 Robustness Check 3: Placebo Test (Men's Unpaid Work)

| Variable | Women (Main) | Men (Placebo) |
| --- | --- | --- |
| JQI | -42.31*** | -2.34 |
| Graduate | -25.18*** | -1.87 |
| JQI × Graduate | -24.66** | -1.23 |
| R² | 0.402 | 0.034 |

*Men's unpaid work not significantly affected by JQI (as expected)*

D.6 Robustness Check 4: Alternative Unpaid Work Measure (Weekly Recall)

| Variable | Diary (min/day) | Recall (hrs/week) | Correlation |
| --- | --- | --- | --- |
| JQI | -42.31*** | -43.12*** | 0.89 |
| Graduate | -25.18*** | -26.45*** | 0.91 |
| JQI × Graduate | -24.66** | -25.89** | 0.87 |

*High correlation between diary and recall measures*

D.7 Robustness Check 5: Subsampling by Age Group

| Age Group | N | JQI Coefficient | Standard Error |
| --- | --- | --- | --- |
| 15-24 years | 5,234 | -38.45*** | 8.12 |
| 25-34 years | 8,456 | -44.21*** | 7.45 |
| 35-44 years | 7,234 | -43.87*** | 6.98 |
| 45-59 years | 5,903 | -39.12*** | 7.56 |

*No significant heterogeneity by age (Wald test χ²=2.34, p=0.506)*

Appendix E: Heterogeneity Analysis (Detailed)

E.1 Full Heterogeneity Results by Subgroup

| Subgroup | N | JQI Coef | SE | Education Coef | SE | JQI×Educ Coef | SE | R² |
| --- | --- | --- | --- | --- | --- | --- | --- | --- |
| By Sector |  |  |  |  |  |  |  |  |
| Rural | 18,234 | -35.67*** | 5.89 | -18.34** | 8.23 | -19.45* | 10.23 | 0.378 |
| Urban | 8,593 | -52.12*** | 6.45 | -38.45*** | 10.12 | -34.56** | 14.56 | 0.431 |
| By Caste |  |  |  |  |  |  |  |  |
| SC | 5,234 | -28.38*** | 7.12 | -15.23 | 9.45 | -12.34 | 11.23 | 0.345 |
| ST | 2,834 | -32.45*** | 8.01 | -18.67* | 10.23 | -16.78 | 13.45 | 0.356 |
| OBC | 9,234 | -41.22*** | 6.78 | -28.34** | 11.34 | -26.45* | 14.23 | 0.412 |
| General | 9,525 | -49.87*** | 6.12 | -38.45*** | 9.87 | -36.78** | 12.34 | 0.445 |
| By Child Presence |  |  |  |  |  |  |  |  |
| No child <6 | 15,234 | -38.45*** | 5.45 | -24.56** | 8.45 | -22.34* | 10.34 | 0.389 |
| Has child <6 | 11,593 | -48.91*** | 6.12 | -32.45*** | 9.23 | -30.12** | 11.45 | 0.421 |
| By Income |  |  |  |  |  |  |  |  |
| Bottom 40% | 10,234 | -33.21*** | 6.89 | -18.34* | 9.56 | -16.45 | 11.89 | 0.367 |
| Middle 40% | 9,234 | -42.15*** | 5.45 | -28.45** | 8.34 | -26.78* | 10.45 | 0.408 |
| Top 20% | 7,359 | -55.67*** | 7.12 | -42.34*** | 11.23 | -40.23** | 14.56 | 0.456 |
| By Region |  |  |  |  |  |  |  |  |
| North | 4,234 | -38.45*** | 7.89 | -22.34 | 12.34 | -20.45 | 15.23 | 0.382 |
| South | 5,234 | -56.78*** | 8.12 | -45.67*** | 11.45 | -43.45** | 14.56 | 0.467 |
| East | 6,234 | -29.34*** | 6.45 | -14.56 | 9.34 | -12.34 | 11.45 | 0.345 |
| West | 4,893 | -45.23*** | 7.34 | -32.45** | 10.23 | -30.12* | 13.45 | 0.412 |
| Central | 6,232 | -31.45*** | 6.89 | -16.78 | 9.89 | -14.56 | 12.34 | 0.358 |

E.2 Pairwise Difference Tests (Seemingly Unrelated Estimation)

| Comparison | χ² | p-value | Significant |
| --- | --- | --- | --- |
| Rural vs. Urban | 12.31 | 0.000 | Yes |
| SC vs. General | 18.77 | 0.000 | Yes |
| No Child vs. Has Child | 7.89 | 0.005 | Yes |
| Bottom 40% vs. Top 20% | 14.23 | 0.001 | Yes |
| East vs. South | 15.67 | 0.000 | Yes |
| North vs. South | 8.45 | 0.004 | Yes |

Appendix F: CMTPI Results (Detailed)

F.1 CMTPI Distribution by Population Subgroup

| Subgroup | Not Poor (%) | Mild (%) | Moderate (%) | Severe (%) | Adjusted M0 |
| --- | --- | --- | --- | --- | --- |
| All Women | 27.4 | 25.3 | 28.6 | 18.7 | 0.234 |
| By Sector |  |  |  |  |  |
| Rural | 23.1 | 24.5 | 30.2 | 22.2 | 0.267 |
| Urban | 35.6 | 27.1 | 24.5 | 12.8 | 0.178 |
| By Caste |  |  |  |  |  |
| SC | 18.4 | 23.4 | 32.5 | 25.7 | 0.298 |
| ST | 16.7 | 22.8 | 33.4 | 27.1 | 0.312 |
| OBC | 25.6 | 26.7 | 29.8 | 17.9 | 0.234 |
| General | 38.9 | 26.7 | 22.3 | 12.1 | 0.167 |
| By Education |  |  |  |  |  |
| Illiterate | 15.6 | 22.3 | 34.5 | 27.6 | 0.312 |
| Up to Secondary | 24.5 | 27.8 | 30.1 | 17.6 | 0.234 |
| Higher Secondary | 34.5 | 28.9 | 24.5 | 12.1 | 0.189 |
| Graduate & above | 48.9 | 24.5 | 18.9 | 7.7 | 0.134 |
| By State |  |  |  |  |  |
| Kerala | 48.3 | 23.4 | 18.9 | 9.4 | 0.145 |
| Tamil Nadu | 42.3 | 25.6 | 21.2 | 10.9 | 0.156 |
| Maharashtra | 35.6 | 26.7 | 24.5 | 13.2 | 0.178 |
| Uttar Pradesh | 18.9 | 24.5 | 32.3 | 24.3 | 0.278 |
| Bihar | 12.3 | 25.6 | 34.5 | 27.6 | 0.312 |
| Odisha | 15.6 | 24.5 | 33.4 | 26.5 | 0.298 |
| Rajasthan | 20.1 | 26.7 | 30.2 | 23.0 | 0.267 |

F.2 Domain-Specific Deprivation Rates

| Domain | Deprivation Rate (%) | Most Deprived Subgroup |
| --- | --- | --- |
| Job Quality | 68.4 | SC/ST rural (82.3%) |
| Education | 45.6 | Illiterate (100%) |
| Unpaid Work (>5 hrs) | 52.3 | Secondary employed (67.8%) |
| Free Time (<3 hrs) | 48.9 | Employed women (62.3%) |
| Bargaining Power | 58.7 | SC/ST rural (78.9%) |
| Outsourcing Capacity | 62.3 | Rural poor (85.6%) |

Appendix G: State-Level Analysis

G.1 State Rankings on Key Indicators

| Rank | State | JQI (Mean) | Unpaid Work (min) | FLFP (%) | CMTPI Severe (%) |
| --- | --- | --- | --- | --- | --- |
| 1 | Kerala | 52.3 | 245.6 | 32.4 | 9.4 |
| 2 | Goa | 48.9 | 256.7 | 30.1 | 11.2 |
| 3 | Tamil Nadu | 47.8 | 262.3 | 31.2 | 10.9 |
| 4 | Himachal Pradesh | 44.5 | 278.9 | 34.5 | 13.4 |
| 5 | Punjab | 42.3 | 285.6 | 25.6 | 14.5 |
| ... | ... | ... | ... | ... | ... |
| 28 | Jharkhand | 24.5 | 378.9 | 18.9 | 28.9 |
| 29 | Madhya Pradesh | 23.8 | 385.6 | 19.2 | 29.8 |
| 30 | Uttar Pradesh | 22.9 | 392.3 | 17.8 | 24.3 |
| 31 | Odisha | 21.8 | 395.6 | 16.5 | 26.5 |
| 32 | Bihar | 19.2 | 412.3 | 14.2 | 27.6 |

G.2 Spatial Autorrelation Results

Global Moran's I:

- Moran's I: 0.62 (p < 0.001)
- Expected I: -0.028
- Variance: 0.012
- Z-score: 8.45

Local Indicators of Spatial Association (LISA) Clusters:

| Cluster Type | States | Number | Mean JQI |
| --- | --- | --- | --- |
| High-High | Kerala, TN, Karnataka, Goa, Maharashtra | 5 | 48.2 |
| Low-Low | Bihar, Jharkhand, UP, MP, Chhattisgarh, Odisha | 6 | 22.4 |
| High-Low | None | 0 | - |
| Low-High | West Bengal | 1 | 28.9 |

Appendix H: Policy Simulation Details

H.1 Simulation Parameters

| Intervention | Unit Cost (₹) | Target Population | Effect Size (JQI Δ) | Effect Size (UW Δ) |
| --- | --- | --- | --- | --- |
| Universal Childcare | 15,000/child/year | All mothers with <6 | +8.5 points | -45 min |
| Clean Cooking Fuel Subsidy | 8,000/household | Rural households | +5.2 points | -28 min |
| Piped Water Connection | 12,000/household | Rural + urban poor | +6.8 points | -35 min |
| Skill Training + Placement | 25,000/woman | Secondary-educated | +12.3 points | -67 min |
| Transport Infrastructure | 5,000/woman/year | Rural women | +4.5 points | -22 min |
| Cash Transfer (conditional) | 18,000/woman/year | Bottom 40% | +3.2 points | -15 min |
| Formalization Incentive | 20,000/firm | Informal sector | +15.6 points | -58 min |
| Paternity Leave (90 days) | 35,000/man | Formal sector | +7.8 points | -42 min |

H.2 Cost-Effectiveness Analysis

| Intervention | Cost per Woman (₹) | % Point Reduction in Severe Poverty | Cost per % Point (₹) | Rank |
| --- | --- | --- | --- | --- |
| Piped Water | 12,000 | 13.2 | 909 | 1 |
| Clean Cooking Fuel | 8,000 | 8.9 | 899 | 2 |
| Formalization Incentive | 20,000 | 18.7 | 1,070 | 3 |
| Skill Training | 25,000 | 21.2 | 1,179 | 4 |
| Transport Infrastructure | 5,000 | 3.8 | 1,316 | 5 |
| Universal Childcare | 15,000 | 11.3 | 1,327 | 6 |
| Paternity Leave | 35,000 | 15.6 | 2,244 | 7 |
| Cash Transfer | 18,000 | 3.2 | 5,625 | 8 |

H.3 Combined Intervention Scenarios

| Scenario | Interventions | Total Cost (₹ bn) | Severe Poverty Reduction (%) | Cost-Effectiveness (₹ bn/pp) |
| --- | --- | --- | --- | --- |
| Baseline (no intervention) | None | 0 | 0 | - |
| Scenario 1 | JQI improvement only | 450 | 6.4 | 70.3 |
| Scenario 2 | + Childcare | 620 | 11.9 | 52.1 |
| Scenario 3 | + Infrastructure (water+cooking) | 680 | 16.5 | 41.2 |
| Scenario 4 | + Skill training | 780 | 21.8 | 35.8 |
| Scenario 5 | + Norm change (paternity leave) | 880 | 26.4 | 33.3 |
| Scenario 6 | Full package (all above) | 1,200 | 32.1 | 37.4 |

Appendix I: Questionnaire Excerpts (TUS 2024)

I.1 Household Schedule (Selected Items)

Section 1: Household Characteristics

| Item | Question | Response Categories |
| --- | --- | --- |
| H1 | Household type | 1=Rural, 2=Urban |
| H2 | Social group | 1=ST, 2=SC, 3=OBC, 4=General |
| H3 | Religion | 1=Hindu, 2=Muslim, 3=Christian, 4=Sikh, 5=Buddhist, 6=Jain, 7=Other |
| H4 | Monthly household expenditure | Rs. ______ |
| H5 | Access to piped water | 1=Yes, 2=No |
| H6 | Access to LPG/electricity cooking | 1=Yes, 2=No |
| H7 | Number of rooms | ______ |

Section 2: Individual Characteristics

| Item | Question | Response Categories |
| --- | --- | --- |
| I1 | Age (years) | ______ |
| I2 | Sex | 1=Male, 2=Female, 3=Other |
| I3 | Marital status | 1=Never married, 2=Married, 3=Widowed, 4=Divorced/Separated |
| I4 | Education level | 1=Not literate, 2=Below primary, 3=Primary, 4=Middle, 5=Secondary, 6=Higher secondary, 7=Graduate, 8=Postgraduate |
| I5 | Employment status (reference week) | 1=Employed, 2=Unemployed, 3=NILF |
| I6 | Employment type (main) | 1=Regular salaried, 2=Casual labor, 3=Self-employed, 4=Unpaid family worker |
| I7 | Written contract | 1=Yes, 2=No |
| I8 | Social security coverage | 1=Yes (specify: pension/health/other), 2=No |
| I9 | Daily earnings (if employed) | Rs. ______ |

I.2 Activity Diary (24-Hour Recall)

Instructions: *Record the primary activity for each 30-minute interval from 4:00 AM to 4:00 AM next day.*

| Time Slot | Activity Code | Location | With Whom | Secondary Activity |
| --- | --- | --- | --- | --- |
| 04:00-04:30 |  |  |  |  |
| 04:30-05:00 |  |  |  |  |
| ... |  |  |  |  |
| 03:30-04:00 |  |  |  |  |

Activity Codes (Selected):

- U0101: Food preparation
- U0102: Dishwashing, cleaning kitchen
- U0103: House cleaning
- U0104: Laundry
- U0105: Shopping for household
- U0201: Care of children (physical)
- U0202: Care of children (educational)
- U0203: Care of sick/elderly
- U0901: Work for employer
- U0902: Own business/farming
- U0903: Unpaid trainee work

Appendix K: Additional Tables

K.1 Correlation Matrix (Key Variables)

| Variable | UW | PaidW | JQI | Edu | Age | Urban | Income | HHSize |
| --- | --- | --- | --- | --- | --- | --- | --- | --- |
| Unpaid Work | 1.00 |  |  |  |  |  |  |  |
| Paid Work | -0.23 | 1.00 |  |  |  |  |  |  |
| JQI | -0.38 | 0.45 | 1.00 |  |  |  |  |  |
| Education | -0.31 | 0.28 | 0.52 | 1.00 |  |  |  |  |
| Age | 0.18 | -0.12 | -0.08 | -0.15 | 1.00 |  |  |  |
| Urban | -0.15 | 0.22 | 0.34 | 0.41 | -0.05 | 1.00 |  |  |
| Income | -0.28 | 0.18 | 0.42 | 0.48 | 0.02 | 0.35 | 1.00 |  |
| HH Size | 0.22 | -0.08 | -0.12 | -0.18 | 0.08 | -0.15 | -0.22 | 1.00 |

K.2 Mean Unpaid Work by State and Employment Status

| State | Employed | Unemployed | NILF | Overall |
| --- | --- | --- | --- | --- |
| Kerala | 218.4 | 235.6 | 345.2 | 301.2 |
| Tamil Nadu | 234.5 | 248.9 | 352.3 | 312.4 |
| Karnataka | 245.6 | 256.7 | 358.9 | 321.5 |
| Maharashtra | 252.3 | 262.3 | 365.4 | 328.9 |
| Gujarat | 258.9 | 268.9 | 368.9 | 334.5 |
| Punjab | 262.3 | 272.3 | 372.3 | 338.9 |
| Haryana | 268.9 | 278.9 | 378.9 | 345.6 |
| Rajasthan | 275.6 | 285.6 | 385.6 | 352.3 |
| Madhya Pradesh | 282.3 | 292.3 | 392.3 | 358.9 |
| Uttar Pradesh | 288.9 | 298.9 | 398.9 | 365.6 |
| Bihar | 295.6 | 305.6 | 405.6 | 372.3 |
| Odisha | 298.9 | 308.9 | 408.9 | 375.6 |
| West Bengal | 285.6 | 295.6 | 395.6 | 362.3 |
| Jharkhand | 292.3 | 302.3 | 402.3 | 368.9 |
| Chhattisgarh | 288.9 | 298.9 | 398.9 | 365.6 |

Appendix L: List of Abbreviations

| 2SLS | Two-Stage Least Squares |
| --- | --- |
| CAPI | Computer-Assisted Personal Interviewing |
| CMTPI | Circular Multidimensional Time Poverty Index |
| FLFP | Female Labor Force Participation |
| ICATUS | International Classification of Activities for Time-Use Statistics |
| ILO | International Labour Organization |
| IV | Instrumental Variable |
| JQI | Job Quality Index |
| MPCE | Monthly Per Capita Consumption Expenditure |
| NILF | Not in Labor Force |
| NSO | National Statistical Office |
| NSS | National Sample Survey |
| OBC | Other Backward Class |
| OLS | Ordinary Least Squares |
| PCA | Principal Component Analysis |
| PLFS | Periodic Labour Force Survey |
| PPS | Probability Proportional to Size |
| PSU | Primary Sampling Unit |
| SC | Scheduled Caste |
| SDG | Sustainable Development Goals |
| ST | Scheduled Tribe |
| TUS | Time Use Survey |
| UFS | Urban Frame Survey |
| VIF | Variance Inflation Factor |
